# Social media responses to the approval of lecanemab in Japan: sentiment analysis

**DOI:** 10.1002/alz.087843

**Published:** 2025-01-09

**Authors:** Kenichiro Sato, Yoshiki Niimi, Ryoko Ihara, Atsushi Iwata, Tetsuaki Arai, Takeshi Iwatsubo

**Affiliations:** ^1^ The University of Tokyo, Tokyo Japan; ^2^ Tokyo Metropolitan Institute for Geriatrics and Gerontology, Tokyo Japan; ^3^ Faculty of Medicine, University of Tsukuba, Tsukuba Japan

## Abstract

**Background:**

Lecanemab, an anti‐amyloid therapy for early Alzheimer’s disease (AD), received full FDA approval in July 2023 and subsequent approval in Japan in September 2023. Public reaction on social media was mixed, reflecting opinions on its efficacy, safety, and cost. We aimed to evaluate public perception of lecanemab’s approval on social media, aiming to inform future communications and development strategies for anti‐amyloid treatments.

**Method:**

We analyzed Japanese‐language social media posts from X (formerly Twitter) and Facebook, collected within two weeks of lecanemab’s approval. Using GPT‐4, we assessed sentiments towards the drug, focusing on efficacy, adverse effects, societal impact, and overall impression.

**Result:**

Out of 478 posts, with 93.3% from X, sentiment analysis showed 43.7% negative, 26.6% neutral, and 29.7% positive overall impressions, with a significant difference by platform (*p = 0.021*). The predominant concern in negative posts was societal impact. There was moderate to good agreement between GPT‐4 and human ratings (kappa > 0.4) for all categories. Specific patterns of attitudes were observed depending on the overall impression to lecanemab’s approval. Medical professionals significantly expressed negative impressions (odds ratio = 1.679).

**Conclusion:**

Responses to lecanemab’s approval displayed specific patterns, emphasizing the need for targeted communication and research direction on anti‐amyloid therapies.

